# The challenging management of Rift Valley Fever in humans: literature review of the clinical disease and algorithm proposal

**DOI:** 10.1186/s12941-020-0346-5

**Published:** 2020-01-22

**Authors:** Emilie Javelle, Alexandre Lesueur, Vincent Pommier de Santi, Franck de Laval, Thibault Lefebvre, Guillaume Holweck, Guillaume André Durand, Isabelle Leparc-Goffart, Gaëtan Texier, Fabrice Simon

**Affiliations:** 1 Laveran Military Teaching Hospital, CS500413384, Marseille Cedex 13, France; 2 IRD, AP-HM, SSA, VITROME, IHU-Méditerranée Infection, Aix Marseille Univ, Marseille, France; 3 French Armed Forces Centre for Epidemiology and Public Health (CESPA), Marseille, France; 4 INSERM, IRD, SESSTIM, Sciences Economiques & Sociales de la Santé & Traitement de l’Information Médicale, Aix Marseille Univ, Marseille, France; 5 French Military Health Service, RSMA Medical Unit, Paris, Mayotte France; 6 French Armed Forces Biomedical Research Institute (IRBA)-CNR des arbovirus-IHU Méditerranée Infection, Marseille, France; 7 IRD 190, Inserm 1207, IHU Méditerranée Infection, AP-HM, UVE, Aix-Marseille Univ, Marseille, France

**Keywords:** Rift Valley Fever, Human disease, Management, Algorithm

## Abstract

Rift Valley Fever (RVF) is an emerging zoonotic arbovirus with a complex cycle of transmission that makes difficult the prediction of its expansion. Recent outbreaks outside Africa have led to rediscover the human disease but it remains poorly known. The wide spectrum of acute and delayed manifestations with potential unfavorable outcome much complicate the management of suspected cases and prediction of morbidity and mortality during an outbreak. We reviewed literature data on bio-clinical characteristics and treatments of RVF human illness. We identified gaps in the field and provided a practical algorithm to assist clinicians in the cases assessment, determination of setting of care and prolonged follow-up.

## Background

Rift Valley Fever virus (RVFV) is an arbovirus, mainly transmitted by mosquitoes, responsible for a zoonosis disease that affects cattle, sheep, camels and goats. It was first identified in 1931 during an investigation into an epidemic among sheep on a farm in the Rift Valley of Kenya [1]. The virus infects also humans through inoculation after contact with infected animals or through ingestion of unpasteurized or uncooked by-products of infected animals, or also through inhalation of aerosols produced during the slaughter of infected animals. However, human infections occurred also from the bites of infected mosquitoes, mainly *Aedes* and *Culex* but also *Anopheles* or *Mansonia*, and other blood-feeding vectors such as flies and ticks have been identified [2–4]. To date, no human-to-human transmission of RVFV has been documented.

RVFV belongs to the *Phenuiviridae family* (*formerly Bunyaviridae*), member of the phlebovirus genus. The enveloped virion contains a tripartite, predominantly negative-sense, single-stranded RNA genome, which codes for structural and non-structural proteins the virus needs to replicate both in mammalian hosts and insect vectors. RVFV attach to cells via the interaction between the viral structural proteins Gn and Gc and C-type lectins, DC-SIGN and I-SIGN [5]. Cells become infected with RVFV by receptor-mediated endocytosis, followed by pH-mediated fusion of virus-endosomal membranes to release nucleocapsids into the cell cytoplasm. Transcription, translation, and genome replication occur in the cytoplasm. The non-structural protein NSs is known to be a major virulence factor allowing the virus to escape host innate immune response. Only one serotype is recognized but strains exist of variable virulence. Moreover, RVFV is classified as a Risk Group 3 agent, and biosafety level (BSL)-3 containment requirements are needed to work with the virus in the laboratory [6].

As other arboviral infections including dengue, chikungunya and zika, RVF is emerging worldwide, due to the globalization of arthropod vectors, mainly mosquitoes, which efficiently transmit an increasing number of old, unrecognized and new viruses. Arboviruses pose a major threat of introduction to several continents, including Europe and North America, with the possibility of co-circulation [7]. The widespread presence of competent vectors, the high viral load in infected animals, trade and global travel, all increase the likelihood of RVFV exportation and establishment outside endemic regions [8–10]. Cases have already been imported to Europe and Asia [9, 11, 12] and concerns have raised about its potential to extend to other parts of Asia, Europe [13] and United States [14, 15]. Such an introduction would cause significant losses to the livestock industry and substantial human morbidity and mortality [16]. Clinicians need to consider RVF in the differential diagnosis for febrile illnesses in a suitable context, however manifestations of RVFV in humans are varied and unspecific including hepatitis, encephalitis, hemorrhagic disease, and retinitis with potential dramatic consequences. The overall case fatality rate is estimated from 0.5 to 2% [8, 17], but higher mortality rates were recorded, as for example 18% by the Saudi Health Ministry in 2000 [18], around 22% in East Africa, West Africa, South Africa and Madagascar from 2006 to 2010 [16], and 28% in Tanzania in 2007 [19].

In 2019 RVFV emerged in Mayotte, a French overseas department and region and gave growth to this work [20]. Strategies of RVFV control appeared us challenging because of its complex biological cycle and its multiple routes of transmission to humans [21]. Besides, the wide clinical spectrum over a long-period of time made very difficult the establishment of standard definitions of human cases and recommendations for their management. We conducted a literature review on the RVF clinical disease and treatments in humans. We identified the state and frontiers of knowledge. Lacking guidelines on the RVF human disease, we proposed an algorithm to assist physicians on the field in the evaluation of cases. This algorithm could help and be improved during next epidemics.

## Methods

**Fig. 1:**
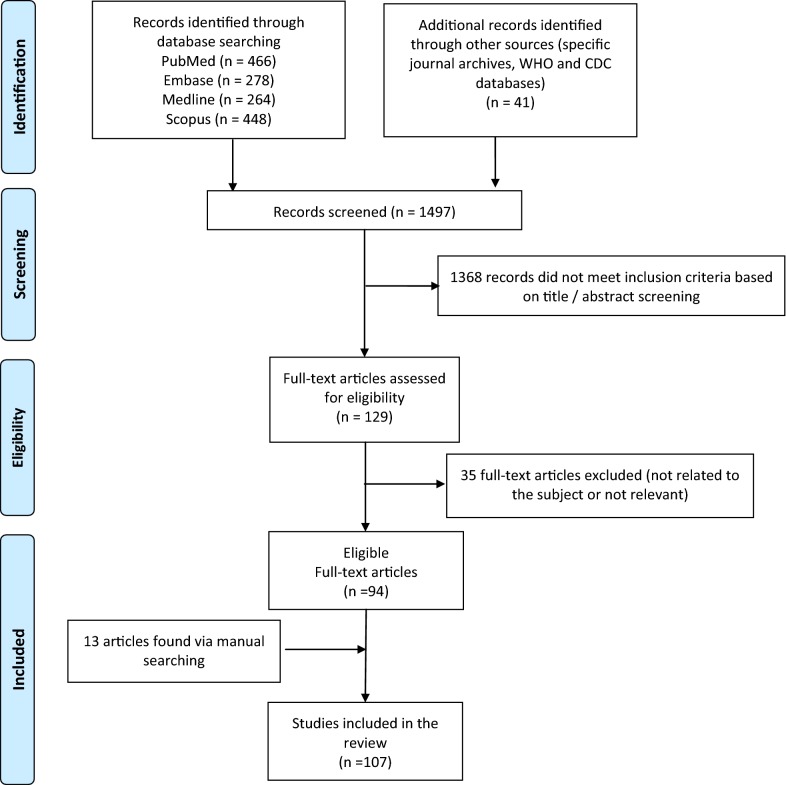
Literature search and study selection

We based on Preferred Reporting Items for Systematic Reviews and Meta-Analyses (PRISMA) guidelines to conduct this clinical review and build the flow diagram (Fig. 1) [22].

We did a literature search for English and French language studies published in electronic databases for an unlimited period until December, 2019: PubMed Central, Embase, Medline and Scopus. We used the keywords “rift valley fever” and “human”. Along with this, we conducted targeted search within the online archives of journals of tropical medicine, which published the first clinical reports of RVF in humans since 1930s, i.e. “South African Medical Journal” and “Transaction of the Royal Society of Tropical Medicine and Hygiene”. We contacted editorial office of other journals to get relevant articles published between 1930s and 1990s. If available, we reviewed citations in PubMed of these first articles in literature. Besides, we consulted the databases, datasets and official reports of the World Health Organization (WHO) and the Centre for Disease Control (CDC) on their official websites and the mentioned references.

We removed duplicates and screened titles and abstracts of all these records to include manuscripts reporting clinical descriptions and/or treatments of RVF in humans (case reports and case series). Animal models for RVFV pathogenicity studies published during the last 10 years were also considered. Serological surveys, works on vaccines, immunology, biology, veterinary science and entomology were excluded.

Totally, 129 articles resulting from these searches with full-text available were assessed for eligibility. Among them, 35 with uncertain RVF diagnosis or without significant content or input were removed.

Relevant references cited in the eligible articles were reviewed and other records were manually searched and added for specific purposes of our article using the following terms “Rift Valley Fever” and “severity”, “severe”, “prognosis”, “death”, “fatal”, “risk factors” and “scores”. At the end, 107 articles were referenced in the final review (Fig. 1).

We used data on RVF human cases reported by the WHO in the rubric “disease outbreak news” [23] and the CDC outbreak summaries [24] to build an epidemiological overview and we used the software Adobe Illustrator 22.1 and macrovector official freepik for figures.

### Epidemiology

**Fig. 2:**
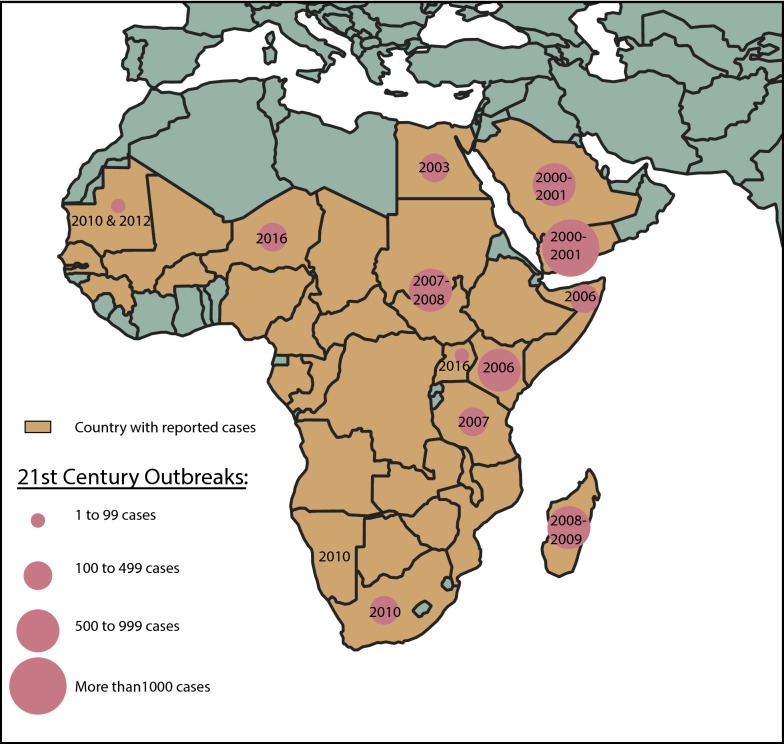
Rift Valley Fever distribution: endemic countries and recent outbreaks since 2000

Human cases have been reported from many African countries following the virus introduction via infected livestock trade [25]. Since the end of 1900s, the virus has extended outside the African continent to Indian Ocean Islands: Madagascar [26], Comoros, and Mayotte [27–29], and has reached the Arabian Peninsula in 2000–2001, with a total estimated 200,000 human infections and 250 deaths in Saudi Arabia and Yemen. During the twenty-first century, outbreaks also occurred in Egypt, Kenya, Somalia, Tanzania, Sudan, Madagascar, the Republic of South Africa, Namibia, Mauritania, Uganda, Niger, and Mayotte (Fig. 2), with fatal cases (Table 1). Recently, RVFV has circulated in Mayotte from November 2018 to August 2019 [30, 31]. Moreover, since September 2019, 365 human cases of RVF have been reported from Sudan, including 11 associated-death (WHO data as of 9 December 2019). The number of secondary cases arising from a single primary case infected with RVFV in an entirely susceptible population, the so-called *R*_*0*_, has been estimated 1.19 with a range including 1 [32, 33], but methods used to calculate this basic reproductive ratio have some limits [34].

**Table 1:** Major Rift Valley Fever outbreaks with cumulated reported human cases and confirmed deaths over the period 2000–2019 (WHO data [23])

Outbreaks dates	Geographic distribution	Reported cases	Cumulated reported cases	Deaths confirmed	Cumulated confirmed deaths
2000–2001	Saudi Arabia, Yemen	886	886	123	123
2006–2007	Kenya	700	1586	158	281
2006–2007	Somalia	114	1700	51	332
2006–2007	Tanzania	264	1964	109	441
2007–2008	Sudan	747	2711	230	671
2008–2009	Madagascar	712	3423	26	697
2009–2011	South Africa and Namibia	> 250	3673	26	723
2010	Mauritania	63	3736	13	736
2012	Mauritania	41	3777	18	754
2013–2014	Mauritania and Senegal	11	3788	0	754
2015	Mauritania	31	3819	8	762
2016–2018	Uganda	16	3835	7	769
2016	Niger	348	4183	33	802
2018	Kenya	26	4209	6	808
2019	Mayotte	143	4352	0	808
2019–…^a^	Sudan^a^	365^a^	4717	11^a^	819

^a^Ongoing outbreak, provisional figures from reports as of 9th December 2019

### The typical benign disease

Infection with RVFV is mostly pauci-symptomatic in humans. General signs may occur in 50 to 95% of infected cases after an incubation period of 2 to 6 days. The typical presentation includes headache, fever, backache and generalized aches in muscles and joints, lasting 4 to 7 days [4, 17, 35, 36]. Malaise, anorexia, nausea, vomiting, flushed face, and conjunctival suffusion were also reported [17, 37]. RVF differs from influenza, dengue and chikungunya as to whether cough, skin involvement (i.e. rash or pruritus) and arthritis are respectively uncommon signs. A slight meningism at the acute stage is not rare, however its prognosis value has never been evaluated [37]. Basically, retro-orbital pains and neck stiffness are features hard to classify because both of them were commonly reported in uncomplicated RVF cases [36], but were also associated with the occurrence of complications [37, 38].

### Complicated and severe expression

Incidences of complications are uncertain because RVFV infection can go unrecognized or be misdiagnosed considering the unspecific symptoms of suspected cases, which overlap with many other co-circulating pathogens [39]. No standard definition of suspected cases exists. Rates of complications measured in studies depend on the definitions and methods of recruitment. Indeed, the use of clinical or biological criteria specific to RVF complications to define suspected cases could lead to underestimate the mild forms [40–42]. Globally, since the first description of the spectrum of RVF in humans by Laughlin et al. during the major outbreak in Egypt in 1977, it is considered that less than 5% of symptomatic cases will present complications including ocular, neurologic and hemorrhagic symptoms, while favorable outcome will occur within 1 week for the others [17]. In this historical series, the different known complications occurred in equal proportions (30–35%), but hepatic or renal failures were not identified. During the 2007 Kenyan outbreak, Kahlon et al. described a clinical syndrome suggestive of severe RVF, characterized by fever, large-joint arthralgia, and gastrointestinal complaints, later followed by jaundice, right upper-quadrant pain, and delirium, often coinciding with hemorrhagic manifestations [43]. Complicated forms could have represented up to 20% of symptomatic cases during recent epidemics [36].

Morbidity, as well as mortality, varied from one to another outbreak. For example, in South Africa in 1975 [44] and in Tanzania in 2007 [19] most of RVF severe cases presented with encephalopathy (respectively 71% and 89%), whereas hemorrhagic manifestations predominated in Mauritania in 2015 (81%) [45] and Madagascar in 2008 (88%) [46]. In Saudi Arabia in 2000, hepatic insufficiency (75%) and renal failures (41%) were the most frequent complications [47]. Moreover, during the epidemic in Madagascar, highly fatal associations of two or more complications were highlighted. These occurred in 11/16 (69%) severe cases, of whom 5 (45%) had encephalitis with hemorrhagic symptoms which were lethal in 2/5 (40%), representing half of the deaths (4/16) [46]. Variations in the RVFV tropism and virulence are hypothesized according to the lineage involved and the possible accumulation of genetic mutations or genomic reassortments [17, 48–50], despite a low overall genomic diversity (∼ 5%) at the nucleotide level [51]. Genetic, ethnic or epidemiologic factors in the population exposed to the virus, as well as access to care also play a role [17, 52].

**Fig. 3:**
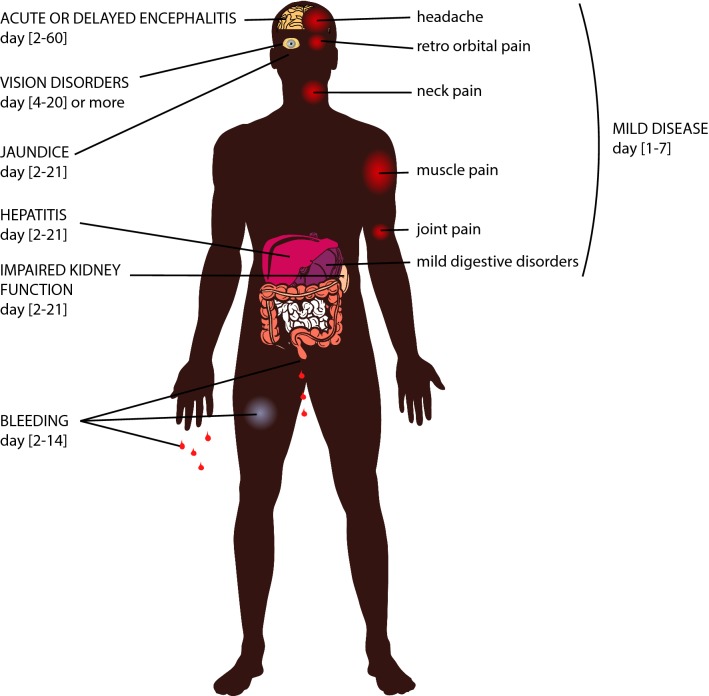
Manifestations of Rift Valley Fever virus infection in humans

Manifestations of RVF in humans are represented in Fig. 3. Alternative diagnoses concern a broad array of conditions which may be worldwide distributed or restricted to endemic areas. Characteristics and differential diagnoses of RVF manifestations are summed-up in Table 2.

**Table 2:** Characteristics and alternative diagnoses of Rift Valley Fever manifestations

RVF manifestations	Time of onset	Frequency	Lethality	Sequelae	Differential infectious diagnoses
Influenza-like syndrome	2 to 6 days of incubation	50–90% of infected people	–	Prolonged asthenia	Arboviruses, influenza virus, HIV (primary infection) *Rickettsia* spp., *Coxiella burnetii*, *Salmonella* spp., bacterial sepsis Malarial parasites
Acute hepatitis	Day 2–21	1–2% of symptomatic cases	High in icteric hepatitis	None	EBV, CMV, HIV, hepatitis viruses A, B, C, D, E, arboviruses (yellow fever, dengue, chikungunya) *Mycoplasma*, *Leptospirosa* spp., *Coxiella burnetii*, *Brucella*, *Bartonella*, *Babesia*, *Pneumococcus*, *Clostridium perfringens* Malarial parasites
Hemorrhagic fever	Day 2–14 (day 2–4)	1–25% of symptomatic cases	25–65%	None	Hemorrhagic viral fevers, *Leptospira* spp.
Ocular signs	Day 4–20	0.5–15% of symptomatic cases	–	Reduced or lost vision	Measles, rubella, influenza, CMV, HSV, VZV, West Nile, chikungunya, dengue and various encephalitis viruses *Rickettsia* spp., *Borrelia burgdorferi*, *Treponema pallidum*
Acute encephalitis	Day 2–10	< 1% of symptomatic cases	50%	Neurologic disorders	Enteroviruses, measles, mumps, rubella, influenza, rabies viruses, arboviruses (West-Nile, dengue, regional encephalitis), HIV *Listeria*, *Rickettsia*, *Treponema pallidum*, *Brucella, Borrelia*, *Leptospirosa* spp., *Chlamydia*, *Mycoplasma*, ±bacterial meningitis (meningococcal, pneumococcal) *Plasmodium falciparum, Trypanosoma* spp., *Toxoplasma gondii*, *Echinococcus*, *Cryptococcus*, *Histoplasma capsulatum*
Delayed encephalitis	Day 4– 60	1–5% of symptomatic cases	< 50%	Neurologic disorders	

*HIV* human immunodeficiency virus, *EBV* Epstein Barr virus, *CMV* cytomegalovirus, *HSV* herpes simplex viruses, *VZV* varicella zoster virus

### Risk factors for severe disease

Determinants for severe RVF outcome are poorly known. A number of retrospective studies suggest that touching, handling, living close to, and consuming animal products are factors associated with increased likelihood of RVF virus infection and possibly more severe outcomes [19, 53]. This is probably linked with a significant exposure to the virus that results in higher inoculation rate. Indeed viremic loads have been reported correlated with severe RVF diseases [54]. Single nucleotide polymorphisms (TLR3, TLR7, TLR8, MyD88, TRIF, MAVS, and RIG-I) were also associated with severe symptomatology [55]. Acute malaria co-occurrence was observed in severe forms and HIV-positive status was associated with a 75% case fatality rate in Tanzania in 2007 [19]. Schistosomal liver co-involvement and bacterial or fungal co-infections were also documented in fatal cases [37].

### Hepatic manifestations

Liver is the primary site of RVFV replication, so that it is frequently early involved during RVFV acute infection [56, 57]. A severe acute hepatotropic disease may occur with liver failure and jaundice within the first 3 weeks of the disease [43]. Tenderness, palpable enlargement and more than threefold elevation in transaminases are criteria of severity [42, 58]. Jaundice was proved to be independently associated with a high mortality rate [40]. Acute hepatitis may complicate with prolonged blood coagulation times and may occur together with or precede fatal hemorrhages or neurologic complications. Autopsy studies and pathogenesis characterization in mouse model found evidence of liver necrosis with RVF viral antigens identified within hepatocytes and Küpffer cells, arguing for a direct virus-induced cellular necrosis [19, 37, 44, 57, 59, 60].

A RVF case with a co-existing condition of cirrhosis after hepatitis B infection died as a result of gastrointestinal bleeding and hepatic encephalitis in Mayotte [28], and 4/31 (13%) severe cases described during the epidemic in Mauritania in 2015 had chronic hepatitis B [61], suggesting that patients with chronic hepatic disorders—mainly hepatitis B chronic infection—could be at higher risk of unfavorable outcome.

### Hemorrhagic fever

Soon after the onset of flu-like illness or acute hepatitis, patients may present bleeding from the nose or gums (gingivorrhagia being a key early warning sign) [62], hematemesis or melaena, petechial/purpuric rash or ecchymoses, menorrhagia, hematuria, or bleeding from venipuncture sites [46, 63]. Yellow fever-like expression were also reported with a first improvement at day 3 followed by a rebound of fever [62]. Epistaxis is not considered a reliable sign of how serious the illness is [64, 65]. Thrombocytopenia is invariably present. Hepato-renal failure with jaundice, disseminated intravascular coagulation and encephalitis can be associated [44, 66]. Overall prevalence is estimated 1%, but prevalence was rather 10% in hospital cohorts [40, 47]. A population-based survey during the 2007 outbreak in Kenya even reported 26% of hemorrhagic RVF disease with a mortality of 23% in this group of cases [67]. Indeed, the mortality rate associated with bleeding manifestations is the highest, up to 65% [40, 68]. Viral load could play an important role in the hemorrhagic expression. In humans studies, it exhibited positive correlation with markers of inflammation (IP-10, CRP, Eotaxin, MCP-2 and Granzyme B), markers of fibrinolysis (tPA and D-dimer), and markers of endothelial function (sICAM-1), but a negative correlation with P-selectin, ADAMTS13, and fibrinogen, which are associated with coagulation pathways occurring on the endothelial surface [69].

### Meningoencephalitis

The onset of meningoencephalitis usually occurs 1 to 4 weeks after the first symptoms (which may be very mild or subclinical), and in some cases neurological complications can manifest beyond 60 days after the initial symptoms of RVF. Clinical features may include intense headache, neurological deficit, rigor, neck rigidity, hyperreflexia, hypersalivation, choreiform movements, loss of memory, hallucinations, confusion, disorientation, vertigo, convulsions, ataxia, lethargy, decerebrate posturing, locked-in syndrome and coma [17, 35, 44, 70–73]. In a human outbreak in Mauritania in 1989, up to 5% of observed infections had encephalitis [71]. Two types of pure encephalitis were described: acute febrile forms with short duration and possibility of death, and subacute forms with a longer duration, a lower fatality rate but frequent sequelae [71]. Pulmonary complications may occur [71, 74], and malaria can worsen the severity of neurologic symptoms [19]. Lethality may be as high as 50% in this form [40].

From Mauritania in 1989, clear a cellular CSF were documented in all cases with encephalitis [71]. In a 18-year old woman treated for chronic myeloid leukemia with acute RVFV encephalitis acquired in Saudi Arabia, CSF was documented predominantly with polynuclear leukocytes. Magnetic resonance imaging (MRI) showed high signal intensity on T2-weighted images in frontoparietal and thalamic regions, with multiple bilateral asymmetrical cortical hyperintense areas consistent with inflammation or ischemia in axial diffusion, while changes in CT-scan of her brain appeared much later [70]. In delayed meningoencephalitis normal glucose and protein concentrations will lymphocytic pleocytosis were found in CSF [17]. In a kidney transplant recipient with cured hepatitis B, presenting acute hepatitis followed by delayed pachymeningitis, specific RVF-IgM were detected in lymphocytic CSF at day 58, while IgG were positive in blood at the first screening at day 44 [74], which was consistent with the first neurologic description in literature [72].

In a mouse model of RVF infection, survivors to primarily hepatitis cleared the virus from liver and blood, but exhibited neuro-invasion and fatal encephalitis [57]. Active viral replication in brain leading to necrotizing encephalitis was documented in several animal models [75, 76]. The route of transmission and prompt robust immune response could be a determining factor of the RVF neurologic disease course [77]. Indeed, whatever the routes of inoculation, RVFV RNA was detected in the brain of infected rats confirming the virus neurotropism [78], but aerosol exposure to RVFV caused earlier and more severe neuropathology in the murine model and fatal encephalitis in primates [75, 76]. In aerosol-infected rats with lethal encephalitis, neutrophils and macrophages were the major cell types infiltrating the CNS, and this was concomitant with microglia activation and extensive cytokine inflammation [78]. Differences in the peripheral blood biomarkers during the course of the neurological disease in African green monkeys were measured with defect in early T-cells, proinflammatory and antiviral responses in lethal encephalitis [79]. Other immune disorders and alteration in vascular permeability in the brain could be more involved in delayed forms [80].

### Other organic failures

During RVFV infection, elevated urea and creatinine levels may be secondary to hypovolemia, multiple-organ dysfunction, or hepatorenal syndrome [39, 81]. Acute hepatonephritis, possibly related to direct RVFV injury, characterized by proteinuria and oliguria were also reported with a bad prognosis [62]. In Mauritania in 2015, creatininemia was meanly more than fourfold upper the reference range in severe cases [61]. In Saudi Arabia in 2000, renal impairment concerned up to 60% of RVF inpatients and dialysis was needed in 90% of them [81]. The mortality rate was 31% in patients with acute renal failure, 25% in those with hepatorenal syndrome, and 31% in patients with primary hepatic involvement and mild renal impairment [81]. Progression to chronic renal failure was not seen [40, 47, 81].

In 2008 in Mayotte, an acute pericarditis with symptoms of right-sided heart failure, relapsing at 1 month, was documented in a 53-year old farmer diagnosed with RVFV infection [28]. In historical *post*-*mortem* examinations, fragmentation in myocardial muscle was found in two cases and RVFV was isolated from one pericardial fluid [37, 44].

### Ocular complications

Macular exudates with potential permanent loss of central visual acuity were firstly described in 7 among 20,000 estimated cases (< 0.05%) during the 1950–51 outbreak in South Africa [82, 83]. The prevalence of ocular manifestations has been estimated to 1% during epidemic outbreaks in Egypt in 1977, and up to 15% both in patients with mild and severe RVF disease during the 2000 outbreak in southwest Saudi Arabia [84]. Unilateral or bilateral symptoms generally occur 5 to 14 days after the RVFV infection, but can be more delayed, and may include decreased visual acuity, scotoma, acute hemorrhagic conjunctivitis and retro-orbital pain [85]. The most frequent and most specific ocular lesion is a macular or paramacular retinitis [86, 87]. The funduscopy by indirect ophthalmoscopy usually shows a single well demarcated necrotic lesion with ill-defined creamy-white patchy lesions of macular retinitis with hemorrhages [84]. The other retinal signs include arterial occlusions, vasculitis (mostly phlebitis and sometimes arteritis) [84], sheathing of the vessels, which are best explored using fluorescein angiography. In series, vitreous reaction with vitreal haze or vitritis occurred in less than one third of patients, optic-nerve head edema or palor were described in 15% of cases with retinal involvement, and no infectious optic neuropathy was reported [84, 85]. Anterior uveitis was associated with a posterior uveitis, defining a panuveitis with aqueous flare and fine non-granulomatous keratic precipitates [84, 85]. Fluorescein angiography performed during the active phase of the disease may show early hypofluorescence with delayed filling of the arterioles and venules, associated with late staining of the lesions [84]. It also helps for the diagnosis of vasculitis, showing vessels sheathing and staining, and vascular occlusions when present. Follow-up fluorescein angiography performed several months after RVF diagnosis have revealed window defect in the area of the retinitis, vascular occlusions and obliterated macular vessels [84]. However, ophthalmoscopic and angiographic features of the RVFV associated retinitis are not specific and can be encountered in several viral or bacterial infections (Table 3) [85, 87–89].

**Table 3:** Main alternative infectious diagnoses for RVFV retinitis and their characteristics

	Retinal lesions	Absent lesions	Localisation	Vitreous lesions	Anterior lesions	Keratic lesions	Onset	Specific treatment
Measles virus	Diffuse retinal edema, small hemorrhages, exudative stellate macular lesions, attenuate vessels, optic disc edema	Retinal necrotic lesions	Diffuse	No	No	Punctuate epithelial erosions and keratitis	Few days after the skin rash or later with SSPE	No
Rubella virus	Chorioretinitis, RPE detachment, bullous retinal detachment	Retinal hemorrhages, vasculitis, optic disc edema	Posterior pole	Mild vitreous reaction	Mild anterior uveitis	Central epithelial keratitis	–	Topical or oral corticosteroids on bullous retinal detachment
Influenza virus	Retinal edema, shiny vesicular dots at the termination of a capillary, small hemorrhages, serous retinal detachment, frosted branch angiitis, neuroretinitis, optic neuritis, uveal effusion	Retinal necrotic lesions	Macular	No	Iridocyclitis	Interstitial keratitis, marginal corneal ulcers	–	Oral corticosteroids for serous retinal detachment, macular edema and frosted branch angiitis
*Borrelia burgdorferi*	Multifocal choroiditis, vasculitis, neuroretinitis, optic disc edema	Retinal necrotic lesions	Diffuse Posterior pole if neuroretinitis	Vitritis	Granulomatous or non-granulomatous anterior uveitis	Stromal keratitis	Several weeks after inoculation	Ceftriaxone Doxycycline Topical or systemic corticosteroids
*Rickettsia* spp.	White retinal lesions adjacent to retinal vessels, serous retinal detachment, retinal sheathing, hemorrhages, vascular leakage or occlusions, optic disc edema, neuroretinitis, optic neuritis, choroidal involvement	Retinal scarring	Adjacent to retinal vessels	Mild to moderate vitreous reaction	No	No	–	Doxycycline Systemic corticosteroids
Dengue virus	Maculopathy with macular edema, focal chorioretinitis, yellow subretinal dots, foveolitis, hemorrhages, venular sheathing, disc edema, vascular occlusion, exudative retinal detachment due to vascular leakage	–	Macular	Vitreous hemorrhages	–	–	1 week after the onset of the fever	No
Chikungunya virus	Multifocal choroiditis, retinal whitening with retinal and macular edema, central retinal artery occlusion, ONH edema when optic neuritis or neuroretinitis, exudative retinal detachment	–	Posterior pole	Mild vitritis	Granulomatous or non granulomatous Anterior uveitis	Keratitis	1 month to 1 year after diagnosis	No
West Nile Virus	Multifocal chorioretinitis, linear clusters of circular deep yellowish lesions, retinal hemorrhages, vascular sheathing, occlusive vasculitis, optic disc edema	–	Midzone, peripheral zone, posterior pole Along retinal nerve fibers	Mild to moderate vitritis	Anterior uveitis	No	Early, contemporary to the fever	No
CMV	Yellow-white areas of necrotizing retinitis, retinal hemorrhages, vascular sheathing, frosted branch angiitis, ONH edema with hemorrhages if optic neuritis	–	Perivascular distribution, central (10%)	Mild vitreous reaction	Mild anterior uveitis	No	–	Ganciclovir Foscarnet
HSV	Retinal edema, ARN syndrome, macular yellowish exudative plaques, flame-shaped hemorrhages, perivenous sheathing, arteriolar narrowing, disc edema, PORN syndrome	–	Macular, diffuse	Mild vitreous reaction	Anterior uveitis with iris atrophy and elevated IOP	Dendritic stromal ulcerations or disciform keratitis	–	Aciclovir
VZV	Focal chorioretinitis with exudates and necrotic lesions, occasional hemorrhages, sheathed vessels, central retinal vein occlusion, ONH edema when optic neuritis, ARN syndrome, PORN syndrome	–	Usually peripheral, possible macular extension	Vitritis when ARN syndrome	Anterior uveitis	Punctuate epithelial keratitis	–	Acyclovir
*Treponema pallidum*	Multifocal chorioretinitis, ASPPC with large pale yellowish subretinal lesion, retinitis, vasculitis (arteritis and phlebitis which can be occlusive), exudative retinal detachment, neuroretinitis	–	Multifocal Posterior pole when ASPPC	Vitritis or vitreous reaction to chorioretinitis	Granulomatous or non- granulomatous anterior uveitis bilateral in 50%	Stromal keratitis	Several weeks after inoculation	Penicillin G Topical or systemic corticosteroids

*RPE* retinal pigment epithelium, *ONH* optic nerve hypoplasia, *ARN syndrome* acute retinal necrosis, *PORN syndrome* progressive outer retinal necrosis, *ASPPC* acute syphilitic posterior placoid chorioretinopathy, *CMV* cytomegalovirus, *HSV* herpes simplex viruses, *VZV* varicella zoster virus, *SSPE* subacute sclerosing panencephalitis

Ocular active lesions resolve spontaneously in 10 to 12 weeks. Macular or paramacular scarring, vascular occlusions and post-infectious optic atrophy associated to the central scarring lead to poor visual acuity outcomes. Retinal complications may cause 40–50% of permanent vision loss, and up to 71% of the affected eyes reached the criteria for legal blindness [38, 84, 85]. No chronic anterior uveitis, posterior synechiae, iris nodules, uveitic glaucoma nor cataract were described [38, 84, 85]. It is not known if the ocular manifestations of the RVF result from direct toxicity of the virus or from an immune response to the infection. Post-mortem examination suggested the presence of focal areas of retinal necrosis and retinal pigment epithelium (RPE) degeneration with round cell inflammatory infiltration and perivascular cuffing but the presence of the virus in the ocular tissues has not been proved. Most of animal models for RVF do not show any ocular disease [35, 57]. In a sheep model quantitative RT-PCR (qRT-PCR) was positive on eye tissues after the viremic phase [90], but retinal complications of RVF could also be caused by antibody-related auto-immune reactions [86].

### Congenital and neonatal infection

In a seroprevalence study, mothers experiencing fetal death or miscarriage had the same RVFV antibody prevalence as those with normal deliveries [91]. A retrospective study in Egypt in 1980 found no increase in the risk of abortion in humans [92]. However a recent cross sectional study has demonstrated an association between infection with RVFV and miscarriage in Sudanese pregnant women (54% versus 12% of risk in non-infected pregnant women with *p *< 0.0001 and OR 7.4 with 95% CI [2.7–20.1] in multiple logistic regression analysis) [93]. The teratogenic potential of the RVFV is unknown. Occasional vertical transmission has been reported, sometimes with fatal outcome in the newborn [94, 95]. Few symptomatic infections were described in pregnant women [91] and children under the age of 10 years old [96, 97]. The question is remaining whether it is the result of a lack of exposure to infected mosquitoes and infected animals, or if there are differences in susceptibility between animals and humans [8].

### Infection prevention and control (IPC) measures

Importantly, hemorrhagic complications require high cautious infection control measures, following the CDC guidance on infection control precautions for hemorrhagic viral fevers (HVFs), while waiting for the exclusion of other HVFs such as Ebola virus disease or Crimean-Congo hemorrhagic fever [39, 98]. Standard precautions with Personal Protective Equipment (PPE) were reported sufficient to prevent from nosocomial transmission of RVFV during the outbreak in Arabian Peninsula [99], and must be implemented according the WHO checklist [100], to care any suspected case regarding the theoretic risk of RVFV transmission through contact with infected blood, tissues, or other body fluids, secretions and excretions. Considering RVF is also mosquito-borne disease [56], we recommend for all-day preventive measures against vectors using physical (long clothing and bed nets), chemical (topical repellents and insecticide impregnations) barriers in the environment of viremic patients. Considering the mean length of the viremia, these measures could be reasonably stopped 1 week after the illness onset, but there is no evidence-based cut-off time to allow donation of blood and removal of tissue or organs for transplantation from a RVFV-infected patient.

### Virologic confirmation of Rift Valley Fever diagnosis

#### Collection of specimens

Samples of suspected cases must be collected with PPE and safe handled adhering to BSL-3 precautions. Specimens must be labelled, packaged in accordance with the guidelines for the transport of dangerous biological goods (triple packaging), stored at 4 °C and addressed to a reference center. If necessary, whole-blood specimens can be dried on blotting paper, stored 30–60 days and transported without refrigeration for retrospective diagnosis confirmation [9].

#### Diagnostic testing

According to WHO a confirmed RVF infection relies on (i) detection of RVFV RNA by reverse transcriptase- polymerase chain reaction (RT-PCR) on sera or plasma; (ii) IgM and IgG detection by enzyme-linked immunosorbent assay (ELISA). Viral isolation is also an assay for laboratory confirmation of RVFV infection but this assay needs to be done on BSL3 and is less sensitive than detection of viral RNA by RT-PCR. Interestingly, RT-PCR for RVFV was reported positive for a prolonged period in urines, semen [74] and whole blood [9]. The RVFV RNA load in blood usually decreases between days 1 to 4 and may be detectable until day 8 after the onset of symptoms [9]. Prolonged and intense viremias were reported during acute encephalitis and hemorrhagic fever. Thus, testing of serial patient specimens collected 24 to 48 h apart may have prognostic value in determining patient outcome. The decrease in viral loads coincides with a rise in RVFV specific IgM and IgG antibodies that may be testing using ELISA. The presence of IgM antibodies appears as an early transient response (day 4 to 60) and protective IgG antibodies persist for several years [36]. A second convalescent blood sample collected 7–14 days after the first is necessary to confirm the seroconversion making a definitive diagnosis of a recent RVF infection.

In case of delayed-onset of encephalitis or ocular complications, imputation to RVFV may be difficult if only IgG are identified in blood at this stage. In human cases with encephalitis, specific IgM and IgG can be detected in CSF [72, 74]. No positive RVFV cultures or RT-PCR have been reported in CSF, or on aqueous or vitreous samples and should be further tested.

### Treatment of RVF cases

Management of RVF human cases comprises IPC measure implementation and general supportive therapy. No specific treatment is currently available. Iatrogenic use of medications such as hepatotoxic analgesics (acetaminophen), aspirin or non-steroid anti-inflammatory drugs, which enhance the risk of hemorrhagic complications, must be avoided in the early stage. Co-infections or alternative diagnosis with parasitic, bacterial, fungal or viral pathogens must be considered and treated as early as possible to improve the outcome. Severe patients should be treated empirically with broad spectrum antibacterial drugs and antimalarial molecules according to the local epidemiology.

In 2000, the Saudi Arabian Ministry of Health evaluated the feasibility of a randomized, placebo-controlled trial using intravenous ribavirin in patients with suspected severe RVF, but no official result was published. In a WHO report of the Emerging and Dangerous Pathogens Laboratory Network in 2016, it was briefly mentioned that ribavirin was used without efficacy in Saudi Arabia [101]. Evidence suggests ribavirin efficacy in animal models [102, 103], but it failed to prevent from neuropathology in mice infected by RVFV by aerosol exposure [75]. Ribavirin is recommended for the treatment and the prophylaxis of hemorrhagic fever due to arenaviruses and bunyaviruses [104] and was successfully used to cure and prevent from Lassa fever [105]. To date, its use is not indicated when RVF diagnosis is confirmed [39, 56]. Antiviral drugs are under development including favipiravir T-705, 2′-fluoro-2′-deoxycytidine (2′-FdC), and benzavir-2 [106–109]. Molecules targeting viral components, host cellular components or pathways, such as the ubiquitin proteasome system, autophagy system, kinases and oxidative stress responses, have demonstrated in vitro efficacy against RVFV [110]. The use of polyclonal immunoglobulins or serum of recovered patients has not been reported. Specific monoclonal neutralizing antibodies could be developed in the coming years [111].

To date, liver transplantation has never been attempted in RVFV fulminant hepatitis. In case reports of encephalitis, the use of amantadine, rifampicin, and dexamethasone [72], doubled prednisone doses with a stop in immunosuppressive drugs [74], and phenytoin [70] were reported but not evaluated precisely. Early renal substitution therapy in patients with severe acute renal failure improve the prognosis and survival [39, 56, 81]. For eye involvement, artificial tear preparations may maintain corneal lubrication and provide temporary comfort for ocular irritation. Topical ophthalmic steroids were used in the RVFV anterior segment manifestations [84]. Aciclovir was used in eyes lesions of other mosquito-transmitted viral disease notably chikungunya, dengue and West Nile (Table 2) [112]. Other antiviral drugs (e.g. ganciclovir, foscarnet) could be administrated through intravitreal routes. In case of elevated intraocular pressure, antiglaucoma medications could be useful. Ocular surgery including cataract removal, retinal hole and detachment repair, vitrectomy, and laser ablation for neovascularization could be additional therapeutics to be evaluated.

### Algorithm proposal for the management of RVF cases based on their severity and complications

**Fig. 4:**
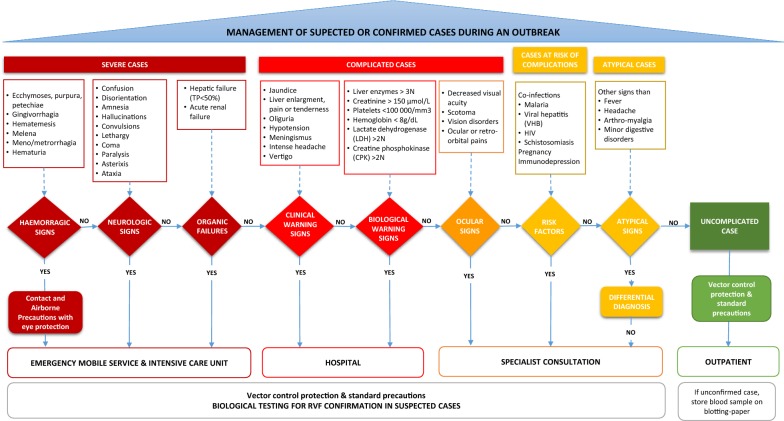
Algorithm to assess and manage Rift Valley Fever cases during an outbreak

Based on the clinical and biological scoring system for the prognosis of RVF established by Adam et al. [64], the CDC definitions of suspected severe RVF cases during the major epidemic in Saudi Arabia in 2000 [42], the updated guidelines for health workers [39], and clinical series in literature [19, 40, 43, 45, 47, 61, 63], we propose an algorithm to help clinicians at the bedside in the classification and referral of patients during a RVF outbreak (Fig. 4). Using the dengue model for case management, we identified clinical and biological warning signs defining complicated cases at risk of severe illness and requiring hospitalization for medical supervision, as we already proposed for chikungunya [113]. Severe illness included hemorrhagic fever, neurological disorders or hepatic/renal failures requiring intensive cares. Ocular signs were classified as complications. Ophthalmologic examination should be prospectively performed in all confirmed cases to detect early asymptomatic RVFV ocular signs and evaluate their potential ability to predict the occurrence of neuro-ophthalmologic complications. This should at least include visual acuity determination, intra-ocular pressure measurement, slit-lamp biomicroscopy and funduscopy by indirect ophthalmoscopy. In case of RVF signs, a fluorescein angiography should be performed as well as fundus photography if available. Indocyanine Green angiography has not been evaluated yet in FVR ocular manifestation but could possibly bring arguments for a choroidal involvement since a delayed peripapillary choroidal filling in the arteriovenous phase of fluorescein angiography has been described [38, 85]. Optic Coherence Tomography (OCT) is a recent technique that is yet to be evaluated in the retinal complications of RVF. OCT could be helpful to describe the retinal lesions and their evolution through time, and could help elucidate the nature of the macular exudate-like lesions described before [114]. We recommend to follow-up RVF patients for at least 1 month after the onset of symptoms to monitor for possible delayed neurological and/or ocular complications. In the lack of adequate medical support, considering the diversity and time course of RVF complications, medical evacuation of confirmed cases may be considered, except if hemorrhages because this presentation is a highly contagious vital emergency.

## Conclusion

Rift Valley Fever has emerged and extended outside Africa in the 2000s, leading to the re-description of the human disease. There is a global concern about the risk of RVFV exportation in areas where competent vectors are present including Europe and North America. Economic losses, human morbidity and mortality may be significant during epidemics. Infection with RVF has a wide clinical spectrum and may result in delayed complications. There are no commercially licensed vaccines nor antiviral treatment for humans. Human cases are often detected when the virus has already spread among livestock and people, hence outbreak control is challenging. Thus, human case surveillance systems for early detection and correct management are essential to reduce global morbidity and mortality. We proposed a tool for physician guidance on the field. This algorithm should be evaluated during ongoing and coming outbreaks, and could help neighboring places in the detection of cases. We identified frontiers of knowledge and remaining uncertainties concerning RVF which deserves more interest. In particular, therapeutic trials on specific supportive care, antiviral molecules or immunotherapies, should be anticipated to be implemented at the start of future epidemics.

## Data Availability

Not applicable.

## Abbreviations

ADAMTS13: a disintegrin and metalloproteinase with thrombospondin motifs
ARN: acute retinal necrosis
ASPPC: acute syphilitic posterior placoid chorioretinopathy
BSL: biosafety level
CDC: Center for Disease Control and Prevention
CI: confidence interval
CMV: cytomegalovirus
CRP: C reactive protein
CSF: cerebrospinal fluid
CT-scan: computed tomography scan
EBV: Epstein Barr virus
ELISA: enzyme-linked immunosorbent assay
HIV: human immunodeficiency virus
HSV: herpes simplex viruses
HVFs: hemorrhagic viral fevers
Ig: immunoglobulin
IPC: infection prevention and control
IP-10: interferon-y induced protein
MAVS: mitochondrial antiviral signaling protein
MCP-2: monocyte chemottractant protein
MRI: magnetic resonance imaging
MyD88: myeloid differentiation
OCT: optic coherence tomography
ONH: optic nerve hypoplasia
PPE: Personal Protective Equipment
PORN: progressive outer retinal necrosis
qRT-PCR: quantitative reverse transcription polymerase chain reaction
RIG-I: retinoic acid-inducible gene
RNA: ribonucleic acid
RPE: retinal pigment epithelium
RVF: Rift Valley Fever
RVFV: Rift Valley Fever virus
sICAM-1: soluble intracellular adhesion molecule
SSPE: subacute sclerosing panencephalitis
TLR8: toll-like receptor
tPA: tissue plasminogen activator
TRIF: TIR-domain-containing adapter-inducing interferon-ß
VZV: varicella zoster virus
WHO: World Health Organization
